# Determinants of health facility utilization for childbirth in rural western Kenya: cross-sectional study

**DOI:** 10.1186/1471-2393-14-265

**Published:** 2014-08-09

**Authors:** Yoshito Kawakatsu, Tomohiko Sugishita, Kennedy Oruenjo, Steve Wakhule, Kennedy Kibosia, Eric Were, Sumihisa Honda

**Affiliations:** JICA SEMAH project, Kisumu, Kenya; Graduate School of Biomedical Sciences, Nagasaki University, Nagasaki, Japan; Ministry of Public Health and Sanitation, Siaya, Kenya; Ministry of Public Health and Sanitation, Kisumu West, Kenya

**Keywords:** Facility delivery, Antenatal care, Determinants, Community health worker, Kenya

## Abstract

**Background:**

Skilled attendance at delivery is recognized as one of the most important factors in preventing maternal death. However, more than 50% of births in Kenya still occur in non-institutional locations supported by family members and/or traditional birth attendants (TBAs). To improve this situation, a study of the determinants of facility delivery, including individual, family and community factors, was necessary to consider effective intervention in Kenya.

**Methods:**

This study was conducted to identify the factors which influence the place of delivery in rural western Kenya, and to recommend ways to improve women’s access to skilled attendants at delivery. A community-based cross-sectional survey was carried out from August to September 2011 in all 64 sub-locations which were covered by community health workers (CHWs). An interviewer-administered questionnaire on seventeen comprehensive variables was administered to 2,560 women who had children aged 12–24 months.

**Results:**

The response rate was 79% (n = 2,026). Of the respondents, 48% of births occurred in a health facility and 52% in a non-institutional location. The significant determinants of facility delivery examined using multivariate analysis were: maternal education level, maternal health knowledge, ANC visits, birth interval, economic status of household, number of household members, household sanitation practices and traveling time to nearest health facility.

**Conclusions:**

The results suggest that the involvement of TBAs to promote facility delivery is still one of the most important strategies. Strengthening CHWs’ performance by focusing on a limited number of topics and clear management guidance might also be an effective intervention. Stressing the importance of regular attendance at ANC (at least four times) would be effective in enhancing motivation for a facility delivery. Based on our findings, those actions to improve the facility delivery rate should focus more on pregnant women who have a low education level, poor health knowledge and short pregnancy spacing. In addition, women with low economic status, a large number of family members and a long distance to travel to a health facility should also be targeted by further interventions.

## Background

More than 500,000 maternal deaths take place annually worldwide. Most of these deaths occur in low- and middle-income countries and could be prevented [1–4]. Research from different countries has shown that the availability and utilization of skilled birth attendants is a key factor in reducing maternal mortality [5]. Skilled attendance at birth is considered one of the most important interventions in preventing maternal death. Skilled birth attendants are defined as "accredited health professional[s] such as a midwife, doctor or nurse who has been educated and trained to proficiency in the skills needed to manage normal (uncomplicated) pregnancies, childbirth and the immediate postnatal period, and in identification, management and referral of complications in women and newborns" [4] which implies deliveries either at home or in health facilities. However, the most effective strategy for mothers living in lower-income countries is to deliver in health facilities under the supervision of health professionals [6].

There is a plethora of evidence to show that higher maternal age [7–10], first birth [11], shorter distance to health facility [12] or availability of transportation [10], and household wealth or ability to pay [6, 7, 13] are significant variables to encourage pregnant women to access skilled delivery. The use of delivery services can also be influenced by family composition, religious or traditional beliefs [7, 12–16] and maternal occupation [17, 18]. Parental education, especially maternal education, is considered one of the strongest factors associated with receiving trained assistance at delivery [8, 11–13]. Antenatal care visits (ANC) encourage pregnant women to deliver at a health facility [7, 19, 20]. In addition, the presence of health workers who provide ANC visits at the community level can also increase the use of skilled attendants [21]. Maternal knowledge about risks of delivery and the availability of health services in their communities is increased by access to information through modern media and maternal/familial education [6, 7]. Moreover, women with a good knowledge of health issues, such as the danger signs during pregnancy, are more likely to deliver in a health facility [22].

In Kenya, 42.6% percent of births occurred in a health facility [23]. The proportion of births assisted by medical personnel increased slightly from 2003 to 2007 [23]. According to Kenya Demographic and Health Survey (KDHS) [23], traditional birth attendants (TBAs) continue to play an important role in childbirth, where they assist with 28 percent of births [23]. Twenty-one percent of births are supported by relatives and friends [23]. The government of Kenya has begun implementing policies to increase deliveries conducted by skilled health personnel. In 1994, the Kenya Health Policy Framework (KHPF) was published to pursue the principles of primary health care. Based on these strategies, community health workers were established to conduct community-based health promotion activities. A concept known as the Community Health Strategy was developed and launched in 2006 as health service provision at Level One. The Strategy was well articulated as one of the flagship programs in the National Health Sector Strategic Plan 2005–2010 (NHPP II), commenced in 2006. The Strategy was revised in 2010 and adopted on a national scale. Identified as Level One of the Kenyan Health System, community health workers (CHWs) in community units (CUs) were required to provide primary health services and to create a demand for the prevention of maternal and childhood deaths. In spite of the government emphasis on facility delivery, there is no research on the determinants of this type of delivery, including the CHWs’ working performance, in Kenya.

The aims of the present study are to assess the current situation of facility delivery, and to investigate factors that influence the utilization of delivery services in health facilities in rural Kenya using a community-based, large-population cross-sectional study.

## Methods

### Research site and population

This study was conducted from August to September, 2011 in all 64 community units covered by community health workers (CHWs), who have been identified as Level One of the health system in Kenya since 2006 [24] and had been volunteering since May, 2011 in Siaya, Ugenya, Gem and Kisumu West districts, Nyanza Province, Kenya. The MOH identified a six-tier health system, in which the Community Unit (level 1) is the proximal implementation unit to promote primary health care services. The range of the population in each CU was from around 2,000 to 10,000 according to the geographical context. The dispensary and health centers (levels 2 and 3 of the Kenyan health system) are engaged in both preventive and curative care. The higher-level hospitals (levels 4, 5 and 6) put more focus on curative and rehabilitative aspects than other levels [25]. The facilities at level 2 and higher provide health services for childbirth. Twenty-four-hour health services are provided at level 3 and above in the Kenyan health system.

CHWs were selected and endorsed by the Community Health Committee, which was democratically elected as a governing body of the Community Unit. Their main activities are door-to-door canvassing to teach health-related preventive methods and collect health-related data from each household. This area is mainly inhabited by subsistence farmers and fishermen. The main ethnic group is Luo and their principal language is the Luo language, followed by Swahili and English. The research population consisted of all mothers who had children aged 12–23 months in this research area.

The sample size was calculated assuming the following points: that 80% power to demonstrate an odds ratio of 1.4 to be significant at the 5% level, if the ratio exposed: unexposed is 1:1 and the prevalence of the outcome is 42.6% in the unexposed. This calculated a sample size of 1,120. In addition to taking the cluster design effect and missing data into consideration, the final sample size was 2,560.

### Study design and data collection

A community-based cross-sectional survey was conducted from August to September, 2011 as a benchmark for the impact assessment of the interventions by the JICA SEMAH project under authorization from MOH, Kenya. A total of 11,906 mothers who had children aged 12–23 months were identified by CHWs in the 64 sub-locations in August, 2011. Forty mothers in each sub-location were selected using random-sampling methods. Finally 2,560 mothers were targeted and were asked, using an interviewer-administered questionnaire, to assess their socio-economic status, their health-seeking behavior and their sanitation practice. Social capital was measured by the standard questionnaire [26]. Most of the variables were mentioned in the study [6]. In addition, the mothers were asked about the number of times CHWs visited their household and their satisfaction score regarding these visits, to generate an indicator of the CHWs’ performance.

The research assistants, not CHWs, were recruited from each sub-location. All were high school graduates (12 years of education) and had previous similar experience of data collection. Furthermore, one day of intensive training, including guidance in data-collection procedures and pre-testing the questionnaire, was conducted.

### Selected study variables

The outcome variable in this study was the place of delivery for pregnant women. While deliveries at any level of health facility (dispensary, health center and hospital or higher level) were considered institutional deliveries, deliveries anywhere other than an institution, including TBA or kinship homes, were considered non-institutional deliveries.

To assess maternal health knowledge, the mothers were asked about the vaccination schedule, danger signs and risk factors in pregnancy and HIV/malaria preventive methods. After scoring by the Clinical Officer, this variable was classified into three levels: low, middle and high. The household wealth index was evaluated using three variables: household assets (e.g. cell phone, television, bicycle, etc.), house materials for the walls, floor and roof, and monthly salary. If they had more than three items out of six household assets, we scored one. If not, we scored zero. If the house materials for the wall, floor and roof were good, we scored one in each variable. This means the maximum score for household materials was three. The monthly salary was also scored in three levels as 0: less than 3,300 KSh; 1: 3,301-5,300 KSh; and 2: higher than 5,300 KSh. The sum of the three variables was calculated and divided into quintiles.

An indicator on the CHWs’ performance was generated by using both frequency of visitation to households and the satisfaction score as reported by the target mothers. Frequency of household visitation was scored as follows:- 0: less than once per month; 1: once per month; 2: more than once per month. Mothers were also asked to specify their level of satisfaction with the CHWs’ performance using a five-point Likert scale, divided as low (0), moderate (1) or high (2). Finally, the variable of the CHWs’ performance was generated by adding the score of the household visitation and the score of satisfaction, and categorized into five quintiles as: poorest (0), poor (1), moderate (2), high (3) and highest (4). Social capital was measured using the standard questionnaire [26]. In this study, social capital consists of two main areas: informal social control, and social cohesion and trust. Each area was represented by a five-point Likert scale. The average score of 10 questions was calculated and classified as: lowest (0), low (1), moderate (2), high (3) and highest (4).

Media and communication equipment was scored by possession of radio, TV and cell phone, and classified as: Possession of one piece of equipment or none (0), two pieces of equipment (1) and all three pieces of equipment (2). In addition, household sanitation practice was scored by using three indicators, i.e. having a toilet, hand-washing facility and a dish rack. They were grouped as: none (0), possession of one out of the three facilities (1), possession of two (2), and possession of all (3).

### Data storage and analysis

Data were verified by a double-entry method and stored using Epi Info version 3.5. Statistical analysis was performed using STATA version 12 (STATA Corporation, TX, USA). The confidence level was set at 95%. Bivariable analyses were conducted in order to assess the association between their delivery place and the community, family, and individual variables. All 17 independent variables were entered into multiple logistic regression analysis and the final model was selected by a backward elimination strategy. The data was weighted with consideration of complex sampling design during the bivariable and multiple logistic regression analysis.

Informed consents from all participants were obtained after full explanation of the study design and purposes. This research was approved by Great Lake University of Kisumu (GLUK) Ethical Review Committee (GERC) in Kenya.

## Results

### Response rate and descriptive statistics

A total of 2,560 mothers who had children aged 12–23 months were visited. Thirty-six mothers refused to participate in this study, 219 mothers were on leave, and the age of 248 children was more than 23 months or less than 12 months. In addition, two children had died before our study started. The data on the place of delivery of 29 mothers was not available. Thus the final analysis was based on 2,026 mothers.

Table 1 presents the general profile of mothers with children aged 12–23 months and is divided into two groups, institutional and non-institutional delivery. Less than half of the mothers (48%) had delivery at a health facility. In the group of mothers who had a facility delivery, 22.6% of the deliveries occurred in a hospital (level 4 or above in the Kenyan health system), 19.3% were in a health center (level 3) and 6.32% were at dispensary level (level 2). Out of the 51% of mothers who delivered in a non-institutional location, 10.1% delivered at a TBA’s place, 13.5% in their home with a TBA, 25.3% in their home with a family member and 2% in other non-institutional locations. Most of the mothers (85%) were married and the education level for about half of them (58%) was primary (elementary) level or higher. Fewer than half (46%) of the mothers had an average health knowledge about danger signs, sanitation practices, etc. More than half of them (55%) had visited health facilities four times or more for ANC services. About one quarter (26%) of the children aged 12–23 months were first-born. As for household characteristics, 21% of them were in the poorest group, 33% were in poor, 22% in middle wealth, 10% of them were in rich and 13% were in our highest group. The total number of household members and children under five were mostly more than five and two respectively. Only 6% of the mothers had no sanitation practice. For 15% it would take more than 60 minutes to go to the nearest health facility on foot and less than 20 minutes for 20% of them. The main means of transportation to get to a health facility was on foot.

**Table 1:** **Socio-demographic characteristics of mothers with children aged 12–23 months and their community variables by place of delivery**

Variables	Institutional N (%)	Non-institutional N (%)	Total N (%)
**Place of delivery**	976 (48.2)	1050 (51.8)	2026 (100)
**Age group (years)**			
< 20	90 (9.2)	76 (7.2)	166 (8.2)
20 – 24	301 (30.8)	340 (32.4)	641 (31.6)
25 – 29	244 (25.0)	246 (23.4)	490 (24.2)
30 – 34	160 (16.4)	159 (15.1)	319 (15.8)
≥ 35	181 (18.6)	229(21.8)	410 (20.2)
**Marital status**			
Single/Divorced/Widowed	153 (15.7)	150 (14.3)	303 (15.0)
Married	823 (84.3)	899 (85.7)	1722 (85.0)
**Education level**			
No education/dropped out from primary	312 (32.0)	530 (50.5)	842 (41.6)
Primary level	421 (43.1)	396 (37.7)	817 (40.3)
Secondary level or higher	243 (24.9)	124 (11.8)	367 (18.1)
**Occupation**			
Subsistence farmer	528 (55.5)	597 (57.3)	1125(56.4)
Domestic worker/student	94 (9.9)	114 (10.9)	208 (10.4)
Other skilled employee	330 (34.7)	331 (31.8)	661 (33.2)
**Literacy in English**			
Illiterate	169 (17.4)	309 (29.4)	478 (23.6)
Literate	804 (82.6)	741 (70.6)	1545 (76.4)
**Maternal health knowledge**			
Low	116 (11.9)	199 (19.0)	315 (15.6)
Middle	426 (43.7)	506 (48.3)	932 (46.1)
High	432 (44.4)	343 (32.7)	775 (38.3)
**ANC visits**			
Less than 4 times	358 (36.7)	544 (51.8)	902 (44.5)
4 times or more	618 (63.3)	506 (48.2)	1124 (55.5)
**Birth interval**			
Short birth interval (<24 months)	30 (3.1)	51 (4.9)	81 (4.0)
Medium birth interval (24–47 months)	78 (8.0)	107 (10.2)	185 (9.1)
Long birth interval (>48 months)	539 (55.2)	690 (65.7)	1229 (60.7)
First birth	329 (33.7)	202 (19.2)	531 (26.2)
**Household wealth index**			
Poorest	154 (16.3)	272 (26.6)	426 (21.7)
Poor	299 (31.6)	350 (34.3)	649 (33.0)
Middle	226 (23.9)	213 (20.9)	439 (22.3)
Rich	110 (11.6)	88 (8.6)	198 (10.1)
Richest	157 (16.6)	98 (9.6)	255 (13.0)
**Media and communication equipment**			
Possession of one item or none	277 (28.4)	416 (39.7)	693 (34.2)
2 items	544 (55.7)	545 (52.0)	1089 (53.8)
All items	155 (15.9)	88 (8.4)	243 (12.0)
**Number of household members**			
5 or more	627 (64.2)	772 (73.5)	1399 (69.1)
Less than 5	349 (35.8)	278 (26.5)	627 (31.0)
**Number of children under five**			
2 or more	584 (59.8)	736 (70.1)	1320 (65.2)
Less than 2	392 (40.2)	314 (29.9)	706 (34.9)
**Household sanitation practice**			
No sanitation facilities	33 (3.4)	95 (9.1)	128 (6.3)
Possession of at least one	320 (32.8)	464 (44.2)	784 (38.7)
Possession of two	319 (32.7)	281 (26.7)	600 (29.6)
Possession of all	304 (31.2)	210 (20.0)	514 (25.4)
**Traveling time to nearest health facility**			
More than 60 min	125 (12.8)	185 (17.6)	310 (15.3)
41 – 60 min	257 (26.3)	304 (29.0)	561 (27.7)
21 – 40 min	349 (35.8)	401 (38.2)	750 (37.0)
20 min or less	245 (25.1)	160 (15.2)	405 (20.0)
**Usual transportation**			
On foot	822 (84.4)	949 (90.5)	1771 (87.5)
Any other means of transportation	152 (15.6)	100 (9.5)	252 (12.5)
**Performance of CHWs**			
Poorest	357 (36.6)	373 (35.6)	730 (36.1)
Poor	82 (8.4)	103 (9.8)	185 (9.2)
Middle	200 (20.5)	193 (18.4)	393 (19.4)
High	182 (18.7)	230 (22.0)	412 (20.4)
Highest	154 (15.8)	148 (14.1)	302 (14.9)
**Social capital**			
Lowest	171 (17.5)	194 (18.5)	365 (18.0)
Low	193 (19.8)	185 (17.6)	378 (18.7)
Middle	229 (23.5)	263 (25.1)	492 (24.3)
High	217 (22.2)	211 (20.1)	428 (21.1)
Highest	166 (17.0)	197 (18.8)	363 (18.0)

### Factors associated with institutional delivery

Table 2 shows factors associated with facility delivery. Compared with younger mothers less than 20 years old, the mothers who were 20–24 and more than 35 years old had significantly lower odds of having a facility delivery. A higher education level, better awareness of health issues and an understanding of English were also significantly associated with facility delivery. If a mother visited four or more times for ANC, she had more than 1.5 times the odds of having an institutional delivery. With first-time pregnancies, the mother had more than twice the odds of delivering in health facilities. In addition, other factors that had a significant effect on facility delivery at the bivariate level were household wealth, possession of media equipment, number in the household and number of children under five, household sanitation practice, distance from the nearest health facility and usual transportation method.

**Table 2:** **Factors associated with institutional delivery at 64 CUs in Kisumu West, Siaya, Ugenya and Gem districts, Nyanza Province, Kenya**

Variables	Bivariable analysis		Multivariate analysis	
	Unadjusted odds ratio (OR)	95% CI	Adjusted OR	95% CI
**Age group (years)**				
< 20	Ref.			
20 – 24	*0.628	0.430-0.917		
25 – 29	0.740	0.502-1.091		
30 – 34	0.735	0.486-1.111		
≥ 35	**0.564	0.380-0.837		
**Marital status**				
Single/Divorced/Widowed	Ref.		Ref.	
Married	0.908	0.695-1.187	1.219	0.892-1.665
**Education level**				
No education/dropped out from primary	Ref		Ref.	
Primary level	***1.886	1.522-2.338	***1.628	1.287-2.058
Secondary level or higher	***3.427	2.567-4.540	***2.354	1.710-3.241
**Occupation**				
Subsistence farmer	Ref.			
Domestic worker/student	1.019	0.746-1.394		
Other skilled employee	1.232	0.995-1.525		
**Literacy in English**				
Illiterate	Ref.			
Literate	***1.984	1.573-2.502		
**Maternal health knowledge**				
Low	Ref.		Ref.	
Middle	1.271	0.956-1.691	1.130	0.829-1.540
High	***1.955	1.458-2.622	**1.539	1.113-2.129
**ANC visits**				
Less than 4 times	Ref.		Ref.	
4 times or more	***1.848	1.523-2.243	***1.660	1.343-2.052
**Birth interval**				
Short birth interval (<24 months)	Ref.		Ref.	
Medium birth interval(24–47 months)	1.234	0.687-2.218	1.475	0.784-2.776
Long birth interval (>48 months)	1.288	0.778-2.131	1.646	0.953-2.844
First birth	**2.426	1.434-4.104	***2.862	1.588-5.158
**Household wealth index**				
Poorest	Ref.		Ref.	
Poor	*1.505	1.143-1.981	*1.420	1.063-1.897
Middle	***2.164	1.608-2.911	***2.097	1.529-2.876
Rich	***2.392	1.629-3.512	**1.948	1.286-2.950
Richest	***3.391	2.382-4.825	***2.290	1.555-3.370
**Media and communication equipment**				
Possession of one item or none	Ref.			
2 items	***1.494	1.207-1.849		
All items	***2.741	1.974-3.807		
**Number of household members**				
5 or more	Ref.		Ref.	
Less than 5	***1.534	1.245-1.890	**1.527	1.190-1.959
**Number of children under five**				
2 or more	Ref.		Ref.	
Less than 2	***1.482	1.210-1.815	1.137	0.887-1.457
**Household sanitation practice**				
No sanitation facilities	Ref.		Ref.	
Possession of at least one	**1.879	1.191-2.964	1.602	0.984-2.606
Possession of two	***3.015	1.895-4.798	***2.480	1.511-4.071
Possession of all	***3.904	2.438-6.252	***2.793	1.683-4.636
**Traveling time to nearest health facility**				
More than 60 min	Ref.		Ref.	
41 – 60 min	*1.394	1.021-1.904	1.357	0.968-1.903
21 – 40 min	*1.373	1.023-1.844	1.324	0.961-1.826
20 min or less	***2.560	1.845-3.554	***2.482	1.735-3.549
**Usual transportation**				
On foot	Ref.		Ref.	
Any other means of transportation	***1.836	1.368-2.466	1.379	0.992-1.918
**Performance of CHWs**				
Poorest	Ref.			
Poor	0.776	0.543-1.109		
Middle	1.158	0.884-1.516		
High	0.796	0.610-1.039		
Highest	1.033	0.770-1.387		
**Social capital**				
Lowest	Ref.			
Low	1.119	0.816-1.535		
Middle	1.005	0.754-1.341		
High	1.117	0.825-1.510		
Highest	0.855	0.634-1.153		

*< 0.05.

**< 0.01.

***< 0.001.

We explored the relationship between institutional delivery and 17 independent variables using logistic regression analysis with backward elimination. The results are also presented in Table 2. Women who finished primary (Adjusted Odds ratio (AOR): 1.63 [1.287 – 2.058]) or secondary school and higher (AOR: 2.35 [1.710 – 3.241]) are more likely to give birth at a health facility than women who did not complete primary level or had no formal education. Higher awareness of health issues was the factor that was significantly associated with facility delivery (AOR: 1.54 [1.113 – 2.129]).

In addition, if women visited ANC services four times or more, they were likely to give birth at a health facility (AOR: 1.66 [1.343 – 2.052]). Those who gave birth for the first time (AOR: 2.86 [1.588 – 5.158]) had a greater chance of delivering in a health care setting than women with short birth intervals. Compared with women who lived in the poorest households, women who lived in poor (AOR: 1.42 [1.063 – 1.897]), middle (AOR: 2.10 [1.529 – 2.876]), rich (AOR: 1.95 [1.286 – 2.950]) and richest (AOR: 2.29 [1.555 – 3.370]) groups were more likely to have deliveries at a health facility. Similarly, women who possessed two sanitation facilities (AOR: 2.48 [1.735 – 3.549]) or all the facilities (AOR: 2.79 [1.683 – 4.636]) were more likely to give birth in health settings, compared to women living in households with no sanitation facilities. Moreover, women living with fewer than five household members were more likely to have a facility delivery (AOR: 1.53 [1.190 – 1.959]). Finally, women who live near a health facility (less than 20 minutes’ walk) have 2.5 times higher odds of giving birth in a medical setting than those who live furthest away (AOR: 2.48 [1.735 – 3.549]).

## Discussion

### Health facility utilization for delivery services

This study was conducted to identify the determinants of health facility utilization for deliveries in western Kenya. In this study, 48% of births occurred as institutional deliveries. Out of those, 23% of the deliveries occurred in hospital (level 4 or higher), 19.3% were in a health center (level 3) and 6.3% were in a dispensary (level 2). However, the number of higher-level health facilities available was less than the number of low-level facilities, which means that there were six hospitals (level 4 or higher), 27 health centers (level 3) and 79 dispensaries (level 2) [27]. This may be due to fewer health workers in lower-level facilities and poor health services. In addition, level 2 health facilities are closed after 5 pm, but labor frequently begins at night. On the other hand, 52% of deliveries occurred in non-institutional locations, such as a TBA’s place (10.1%), the mother’s home with a TBA assistant (13.5%), the mother’s home with family and friends (25.3%) and other places (2%). Despite the fact that TBAs are not officially recognized as qualified caregivers, nearly half of the non-institutional deliveries were assisted by TBAs and family members. This result implies that community members still demand the informal health service provision available in their residential areas and rely on conventional kinship and social network.

### Determinants of institutional delivery

The significant determinants of institutional delivery were: maternal education level, maternal health knowledge, ANC visit, birth interval, socio-economic status of the household, family size, household sanitation practices and proximity of the nearest health facility.

Maternal education as one of the significant determinants of institutional delivery has been well-established and evidenced within several studies [6, 28, 29]. Higher levels of education improve maternal knowledge, increase self-confidence and also enhance awareness of available health resources in the community. Female decision-making abilities and problem-solving skills are also strengthened by receiving a higher education and change the household dynamics [8, 28, 30, 31].

Better maternal health knowledge was also a significant determinant of facility delivery in this study. A study found that women in Zambia who can pinpoint the danger signs in pregnancy are more likely to deliver in a health facility than those without such knowledge [22]. High maternal health knowledge may be able to positively influence a woman’s care-seeking behaviors as well as enabling her to recognize the danger signs early. However, this maternal knowledge may be the result of frequent contacts with skilled health personnel; therefore it is necessary to conduct a prospective study.

In addition, receiving antenatal care four times or more was one of the significant positive determinants to improving institutional delivery in this study. Most of the studies have found that women who use ANC services are much more likely to receive skilled attendants at delivery [19, 21, 32]. Women who visit health facilities for ANC services receive more opportunities for health professionals to explain the advantages of skilled attendants and information on the status of their pregnancies. Other indicators or markers of a maternal health-seeking behavior may include ANC attendance or being able to interact with the health system and health facility in a more comfortable manner [6].

Women in their first pregnancy are more likely to have their baby in a health facility [33]. The first birth is known to be more difficult and the family may help the mother to get better care [6]. Contrasting with this is the fact that women of higher parity tend to rely on their previous experiences and may not think of the necessity for health care services if previous deliveries were uncomplicated [34].

Household wealth was also found to be an important determinant of facility delivery. We noted that there was a strong correlation between the wealth index and the use of maternal care services. This is consistent with findings from the previous studies [28]. Households on quite a limited budget could have difficulty paying fees and therefore would tend to be less likely to use a health facility for delivery. In addition, women of low economic status are known to have lower rates of seeking maternal health care. A greater use of services is more commonly seen in households with higher economic status where modern health care services are easily accessible [11].

There is a significant association with family size and facility delivery in this study. Stephen et al. [35] argue that families in the area with high birth rates may be more conservative toward antenatal and maternal health services. A large family size might be a reflection of the degree of a woman’s independence. Additionally, the decision-maker in the household with a large family size is usually the father. Further research should include the indicator on these factors, so that the effect of family size will become a more concrete indication.

In households that have sanitation facilities, women are more likely to give birth at a health facility. This indicator would be associated with health awareness/practices in the household and the level of available expenditure for health amenities.

Finally, a number of studies have examined the effect of distance, quality and transportation means [14, 21, 32]. It is clear that transportation issues are a major factor in the decision to go to a health facility (phase 1 delay). This factor also influences the delay caused by distance from home to the health facility (phase 2 delay). In our study, the mode of travel to the nearest facility was not significantly associated with facility delivery. Most labor began in the late evening, and at that time it was difficult to find the usual transportation in the research area.

In our study, performance of CHWs and social capital as community variables were not significant determinants of institutional delivery. Even though one of the CHWs’ main activities was household visitation to provide health education to community members, there was no effect on facility delivery. A study in Nepal also found that household visitation by health workers was associated with the increased utilization of ANCs and post-natal care. However, this factor did not have a significant impact on skilled birth attendance [36]. Recent evidence from Tanzania suggests that an increase in home visits by health personnel at the grass roots level can increase the use of ANC visits as well as delivery by a skilled birth attendant [37]. The intervention of the study in Tanzania was to train safe motherhood promoters (SMPs) equivalent to CHWs in Kenya. Their roles are focused on promoting early ANCs, complete ANCs and delivery with a skilled attendant. Compared with SMPs in Tanzania, CHWs in Kenya have multiple tasks focusing not only on antenatal care, facility delivery and postnatal care but also case management for the general population, heavy reporting, data management, community entrepreneurship, and so on. It would be better to give CHWs limited, clear objectives and knowledge to promote community health and reduce the amount of reporting duty. This would make the work of CHWs more effective, enhance community awareness and increase utilization of ANC visits and delivery by skilled health personnel. In addition, it would be necessary to develop hands-on tools about the merits of institutional delivery and the risks of non-institutional delivery for the CHWs to work more effectively.

### Strengths and limitations

This study was a large community-based cross-sectional study with adequate power to detect important differences in facility delivery between different groups. Recommended by Sabine Gabrysch et al. [6], seventeen comprehensive individual, households and community factors were assessed to adjust confounding factors.

This study has some limitations that should be noted. Firstly, the participants provided all of the data. There was no way to validate the obtained information with any objective source such as a health facility card. Although self-reporting can be sketchy, it can be assumed that biases are less likely in pregnancy-related events as compared to more sensitive issues, such as sexual behavior and drug abuse. Secondly, we examined the association between current situations and previous places of delivery. This limitation is especially related to CHWs’ status and social capital. To minimize these limitations, it would be better for further research to select pregnant women before their delivery and the use of a prospective study would be needed to examine causal relationship. Although we examined seventeen comprehensive independent variables, we missed some variables in this research, such as parity and time of onset of labor. In addition, it was difficult to get accurate data on the pregnancy or delivery complications in this research. Therefore, it would be better for future research to include these variables and to consider clear definitions of the variables.

## Conclusion

Despite the limitations, the determinants we found have useful implications for both health care providers and decision-makers in health programs. The results suggest that the involvement of TBAs to promote facility delivery is still one of the most effective strategies. Strengthening CHWs’ performance with clear guidance and hands-on tools might also be an effective intervention. Stressing the importance of regular attendance at ANC (more than four times) would be effective to enhance motivation for a facility delivery. Based on our findings, those actions to improve the facility delivery rate should be focused more on those pregnant women who have a low education level and immature health knowledge. In addition, women who have short birth intervals and live in households with poor economic status, a large number of family members and are far from the nearest health facility should also be targeted.
